# PixelBNN: Augmenting the PixelCNN with Batch Normalization and the Presentation of a Fast Architecture for Retinal Vessel Segmentation

**DOI:** 10.3390/jimaging5020026

**Published:** 2019-02-02

**Authors:** Henry A. Leopold, Jeff Orchard, John S. Zelek, Vasudevan Lakshminarayanan

**Affiliations:** 1 Department of Systems Design Engineering, University of Waterloo, Waterloo, ON N2L 3G1, Canada; 2 David R. Cheriton School of Computer Science, University of Waterloo, Waterloo, ON N2L 3G1, Canada

**Keywords:** convolutional networks, deep learning, retinal vessels, image segmentation, ophthalmology, retina, ophthalmic diagnosis

## Abstract

Analysis of retinal fundus images is essential for eye-care physicians in the diagnosis, care and treatment of patients. Accurate fundus and/or retinal vessel maps give rise to longitudinal studies able to utilize multimedia image registration and disease/condition status measurements, as well as applications in surgery preparation and biometrics. The segmentation of retinal morphology has numerous applications in assessing ophthalmologic and cardiovascular disease pathologies. Computer-aided segmentation of the vasculature has proven to be a challenge, mainly due to inconsistencies such as noise and variations in hue and brightness that can greatly reduce the quality of fundus images. The goal of this work is to collate different key performance indicators (KPIs) and state-of-the-art methods applied to this task, frame computational efficiency–performance trade-offs under varying degrees of information loss using common datasets, and introduce PixelBNN, a highly efficient deep method for automating the segmentation of fundus morphologies. The model was trained, tested and cross tested on the DRIVE, STARE and CHASE_DB1 retinal vessel segmentation datasets. Performance was evaluated using G-mean, Mathews Correlation Coefficient and F1-score, with the main success measure being computation speed. The network was 8.5× faster than the current state-of-the-art at test time and performed comparatively well, considering a 5× to 19× reduction in information from resizing images during preprocessing.

## 1. Introduction

The segmentation of retinal morphology has numerous applications in assessing ophthalmologic and cardiovascular disease pathologies, such as Glaucoma and Diabetes [1]. Diabetic retinopathy (DR) is one of the main causes of blindness globally, the severity of which can be rapidly assessed based on retinal vascular structure [2]. Glaucoma, another major cause for global blindness, can be diagnosed based on the properties of the optic nerve head (ONH). Analysis of the ONH typically requires the removal of vasculature for computational methods. Similar analyses of other structures within the eye benefit from the removal of retinal vessels making the segmentation and subtraction of vasculature critical to many forms of fundus analysis. Direct assessment of vessel characteristics such as length, width, tortuosity and branching patterns can uncover abnormal growth patterns or other disease markers—such as the presence of aneurysms, which are used to evaluate the severity of numerous health conditions including diabetes, arteriosclerosis, hypertension, cardiovascular disease and stroke [3]. For these types of diseases, early detection is critical in minimizing the risk complications and vision loss in the case of DR, glaucoma and other conditions of the eye [4]; early detection is often the most effective method for reducing patient risk through modifications to lifestyle, medication and acute monitoring [5]. Similarly, the same information—this time gleaned from youth, can be used as indicators in the prediction of those individuals’ health later in life [6].

Retinal vessel segmentation from fundus images plays a key role in computer-aided retinal analyses, either in the assessment of the vessels themselves or in vessel removal prior to the evaluation of other morphologies, such as the ONH and macula. For this reason, it has been the most crucial step of practically all non-deep computer based analyses of the fundus [7]. Automated computer image analysis provides a robust alternative to direct ophthalmoscopy by a medical specialist, providing opportunities for more comprehensive analysis through techniques such as batch image analysis [8]. As such, much research has gone into automatically measuring retinal morphology, traditionally utilizing images captured via fundus cameras. However, automatic segmentation of the vasculature has proven to be a challenge, mainly due to inconsistencies such as noise or variations in hue and brightness, which can greatly reduce the quality of fundus images [9]. Traditional retinal pathology and morphology segmentation techniques often evaluate the green channel of RGB fundus images, as it is believed to be the “best” channel for assessing vascular tissue and lesions, while the red and blue channels suffer low contrast and high noise [10]. Unfortunately, variations in image quality and patient ethnicity often invalidate this belief in real world settings.

Accurate feature extraction from retinal fundus images is essential for eye-care specialists in the care and treatment of their patients. Unfortunately, experts are often inconsistent in diagnosing retinal health conditions resulting in unnecessary complications [11]. Computer-aided detection (CAD) methods are being utilized for retinal disease evaluation in commercial settings, however most traditional methods are unable to match the performance of clinicians. These systems under-perform due to variations in image properties and quality, resulting from the use of varying capture devices and the experience of the user [9]. To properly build and train an algorithm for commercial settings would require extensive effort by clinicians in the labelling of each and every dataset—a feat that mitigates the value of CAD systems. Overcoming these challenges would give rise to longitudinal studies able to utilize multi-modal image registration and disease/condition status measurements, as well as make applications in surgery preparation and biometrics more viable [9].

The emergence of deep learning methods has enabled the development of CAD systems with an unprecedented ability to generalize across datasets, overcoming the shortcoming of traditional or “shallow” algorithms. Computational methods for image analysis are divided into supervised and unsupervised techniques. Prior to deep learning, supervised methods encompassed pattern recognition algorithms, such as k-nearest neighbours, decision trees and support vector machines (SVMs). Examples of such methods in the segmentation of retinal vessels include 2D Gabor wavelet and Bayesian classifiers [10], line operators and SVMs [3] and AdaBoost-based classifiers [12]. Supervised methods require that training materials be prepared by an expert, traditionally limiting the application of shallow methods. Unsupervised techniques stimulate a response within the pixels of an image to determine class membership and do not require manual delineations. The majority of deep learning approaches fall into the supervised learning category, due to their dependence on ground truths during training. Often, unsupervised deep learning techniques refer to unsupervised pertraining to improving network parameter initialization as well as some generative and adversarial methods.

Deep learning overcomes shallow methods’ inability to generalize across datasets through the random generation and selection of a series of increasingly dimensional feature abstractions from combinations of multiple non-linear transformations on a dataset [13]. Applications of these techniques for object recognition in images first appeared in 2006 during the MNIST digit image classification problem, of which convolutional neural networks (CNNs) currently hold the highest accuracy [14]. Like other deep neural networks (DNNs), CNNs are designed modularly with a series of layers selected to address different classification problems. A layer is comprised of an input, output, size (number of “neurons”) and a varying number of parameters/hyper-parameters that govern its operation. The most common layers include convolutional layers, pooling/subsampling layers and fully connected layers.

A popular method for facilitating multi-resolution generalizability with fully convolutional networks is the use of dilated convolutions within the model [15,16]. Dilated convolutions can be computationally expensive, as they continuously increase in size through the utilization of zero padding to prevent information loss. Downsampling is another family of methods that sample features during strided convolution at one or more intermediate stages of a fully convolutional network (FCN), later fusing the samples during upsampling [17] and/or multi-level classifiers [18]. Such methods take advantage of striding to achieve similar processing improvements as dilated convolutions with increased computational efficiency, albeit with a loss in information. Variations in downsampling methods aim to compensate for this loss of information. Implementing both long and short skip connections has been shown to prevent information loss and increase convergence speed [19], while mitigating losses in performance [20].

Deep algorithms often pose retinal image analysis as a binary classification task, learning to differentiate morphologies based on performance masks manually delineated from the images. The current limitation with most unsupervised methods is that they utilize a set of predefined linear kernels to convolve the images or templates that are sensitive to variations in image quality and fundus morphologies [8]. Deep learning approaches overcome these limitations, and have been shown to outperform shallow methods for screening and other tasks in diagnostic retinopathy [21,22]. A recent review chapter discusses many of these issues and related methodologies [23].

The goal of this work is to collate different key performance indicators (KPIs) and state-of-the-art methods applied to this task, introduce PixelBNN, and frame computational efficiency–performance trade-offs under varying degrees of information loss using common datasets. PixelBNN is a novel variation of PixelCNN [24]—a dense FCN, that takes a fundus image as the input and returns a binary segmentation mask of the same dimension. The network was able to evaluate test images in 0.0466 s, 8.5× faster than the state-of-the-art when using resized images, while retaining comparable performance. Section 2 discusses the method and network architecture. Section 3 describes the experimental design. The resulting network performance is described in Section 4. Lastly, Section 5 discusses the results, future work and then concludes the paper.

## 2. Material and Methods

Deep learning methods for retinal segmentation are typically based on techniques which have been successfully applied to image segmentation in other fields, and often utilize stochastic gradient descent (SGD) to optimize the network [21]. Recent work into stochastic gradient-based optimization has incorporated adaptive estimates of lower-order moments, resulting in the Adam optimization method, which is further described below [25]. Adam was first successfully applied to the problem of retinal vessel segmentation by the authors, laying the foundation for this work [26].

Herein, a fully-residual autoencoder batch normalization network (“PixelBNN”) was trained via a random sampling strategy whereby samples are randomly augmented from a training set of fundus images and fed into the model, as described in Section 2.2. PixelBNN utilizes gated residual convolutional and deconvolutional layers activated by concatenated rectifying linear units (CReLU), similar to PixelCNN [15,24] and PixelCNN++ [27]. PixelBNN differs from its predecessors in three areas; (1) varied convolutional filter streams, (2) gating strategy, and (3) introduction of batch normalization layers [28] from which it draws its name.

### 2.1. Datasets

#### 2.1.1. DRIVE

The CNN was trained and tested against the Digital Retinal Images for Vessel Extraction (DRIVE) database (http://www.isi.uu.nl/Research/Databases/DRIVE/), a standardized set of fundus images used to gauge the effectiveness of classification algorithms [29]. The images were 8 bits per RGBA channel with a 565 × 584 pixel resolution. The data set comprised of 20 training images with manually delineated label masks and 20 test images with two sets of manually delineated label masks by the first and second human observers. The images were collected for a diabetic retinopathy screening program in the Netherlands using a Canon CR5 non-mydriatic 3CCD camera with a 45° field of view [29].

#### 2.1.2. STARE

The Structured Analysis of the Retina database (http://cecas.clemson.edu/~ahoover/stare/) has 400 retinal images which are acquired using TopCon TRV-50 retinal camera with 35° field of view and pixel resolution of 700 × 605. The database was populated and funded through the US National Institutes of Health [1]. A subset of the data was labelled by two experts, thereby providing 20 images with labels and ground truths. To compensate for the small number of images, four-fold cross-validation was used. Therein, the network was trained over four runs, leaving five images out each time, resulting in all 20 images being evaluated without overlapping the training set, thus minimizing network bias.

#### 2.1.3. CHASE_DB1

The third dataset used in this study was a subset of the Child Heart and Health Study in England database (CHASE_DB1), containing 28 paired high-resolution (1280 × 960 pixels) fundus images from each eye of 14 children, captured with a 30° field of view using a Nidek NM-200-D fundus camera. Compared to STARE, CHASE_DB1 is more susceptible to bias as the images are all pairs from the same patient—this restricts the number of samples to 14. Due to this constraint and for the same reasons as with STARE, four-fold cross-validation was used to preclude overlapping datasets between training and test time, this time grouping sets by patients. (https://blogs.kingston.ac.uk/retinal/chasedb1/).

### 2.2. Preprocessing

The most common and effective method for correcting inconsistencies within an image dataset is by comparing the histogram of an image obtained to that of an ideal histogram describing the brightness, contrast and signal/noise ratio, and/or determination of image clarity by assessing morphological features [30]. Fundus images typically contain between 500 × 500 to 2000 × 2000 pixels, making training a classifier a memory and time consuming ordeal. Rather than processing an entire image, the image–label pairs are randomly cropped and resized using bicubic interpolation to 256 × 256 pixels, flipped, rotated and/or enhanced to extend the dataset.

#### 2.2.1. Continuous Pixel Space

It has been shown that a continuous domain representation of pixel colour channels vastly improves memory efficiency during training [31]. This is primarily due to dimensionality reduction from initial channel values to a distribution of (−0.5 to 0.5). features are learned with densely packed gradients rather than needing to keep track of very sparse values associated with typical channel values [27]. Herein, the raw pixel values of each channel were remapped from (0, 255) to (−0.5, 0.5).

#### 2.2.2. Image Enhancement

Local histogram enhancement methods greatly improve image quality and contrast, improving network performance during training and evaluation. Rather than sampling all pixels within an image once, histograms were generated for subsections of the image, each of which is normalized. One limitation for local methods is the risk of enhancing noise within the image. Contrast limited adaptive histogram equalization (CLAHE) is one method that overcomes this limitation. CLAHE limits the maximum pixel intensity peaks within a histogram, redistributing the values across all intensities prior to histogram equalization [32]. This is the contrast enhancement method used herein.

### 2.3. Network Architecture

**Figure 1:**
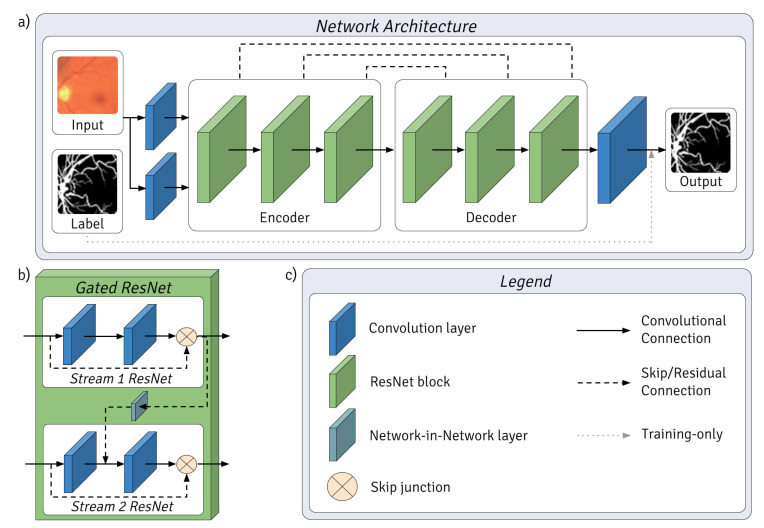
(**a**) The overall network architecture is shown, whereby processed image patches are passed through two convolution layers with different filters to create parallel input streams for the encoder. Convolutional downsampling occurred between each ResNet block in the encoder and deconvolutional upsampling in the decoder. Each ResNet block consisted of 4 Gated ResNets, forming encoder–decoder pairs. The output was a vessel mask of equal size to the input. The label was used to train the network. (**b**) Gated ResNet architecture. (**c**) Legend for the layers, blocks and connections.

PixelBNN is a fully-residual autoencoder with gated residual streams, each initialized by differing convolutional filters. It is based on UNET [33], PixelCNN [15] as well as various work on the use of skip connections and batch normalization within fully convolutional networks [17,18,19,34]. Figure 1a illustrates the architecture of the proposed method, whereby processed image patches were passed through two convolution layers with different filters to create parallel input streams for the encoder. Convolutional downsampling occurred between each ResNet block in the encoder and deconvolutional upsampling in the decoder. Each gated ResNet block consisted of four gated ResNets, each of which had an architecture as shown in Figure 1b. Each gated ResNet was made up of convolution layers with kernel size 3 and stride of 1. Stream 1 ResNet was gated with Stream 2 by a network-in-network (NIN) layer—which is a 1 × 1 convolutional layer like those found in Inception models [20]—which concatenated the output features from the first steam with those of the second. PixelBNN utilized convolutional downsampling with a stride of 2, as well as long and short skip connections. Each gated ResNet in the encoder had a skip connection to a paired Gated ResNet in the decoder. Dropout was applied to outbound connections of each gated ResNet during downsampling. The output was a vessel mask of equal size to the input. The label is used to train the network, specifically to calculate the loss of the generated vessel mask.

This method differs from prior work in the layer architecture by the use of gated filter streams and regularization by batch normalization joint with dropout during training. While nuanced, the network further differentiates from many state-of-the-art architectures in its use of Adam optimization, layer activation by CReLU and use of downsampling in place of other multi-resolution strategies. The network made extensive use of CReLU to reduce feature redundancy and negative information loss that would otherwise be incurred with the use of rectified linear units (ReLU). CReLU models have been shown to consistently outperform ReLU models of equivalent size while reducing the number of parameters by half, leading to significant gains in performance [35]. It differs from PixelCNN++ [27] in three ways. Firstly, feature maps were implemented as with UNET [33] with a starting value of 16, doubling at each downsampling. Secondly, in the use of batch normalization after each downsampling and before dropout, rather than dropout alone. Thirdly, it differs in its use of paired convolution layers on continuous pixel space RGB images.

The architecture was influenced by the human vision system; more detail on this subject is covered in prior work by the authors [23]:

- The use of two parallel input streams resembles bipolar cells in the retina, each stream possessing different yet potentially overlapping feature spaces initialized by different convolutional kernels.
- The layer structure was based on that of the lateral geniculate nucleus, visual cortices (V1, V2) and medial temporal Gyrus, whereby each is represented by an encoder–decoder pair of gated ResNet blocks.
- Final classification was executed by a convolutional layer which concatenates the outputs of the final gated ResNet block, as the inferotemporal cortex is believed to do.

### 2.4. Platform

Training and testing of the proposed method was done using a computer with an Intel(R) Core(TM) i7-5820K CPU with 3.30GHz of processing power, 32 GB of RAM and a GM200 GeForce GTX TITAN X graphics card equivalent to 3072 CUDA cores. On this platform, it took roughly 14 h to train the network. At test time, the network processed a single image in 0.0466 s using the same system. In this study, Tensorflow [36] and other python scientific, imaging, and graphing libraries were used to evaluate the results.

### 2.5. Experiment Design

This paper explores the impact of information loss and computational efficiency due to resizing on the task of vessel segmentation in fundus images. It presents PixelBNN, a novel network architecture for multi-resolution image segmentation and feature extraction based on PixelCNN. This was the first time this family of dense fully connected convolutional networks have been applied to fundus images. The specific task of retinal vessel segmentation was chosen due to the availability of different datasets that together provided ample variances for cross-validation, training efficiency, model performance, and robustness. Architectural elements of the network have been thoroughly evaluated in the literature, as mentioned in Section 2.3. A comparison with the full-resolution datasets will be carried out along side an ablation study, which is beyond the scope of this paper and left for future work. The goal of this work was to collate different KPIs and state-of-the-art methods applied to this task, introduce PixelBNN, and frame computational efficiency–performance trade-offs under varying degrees of information loss using common datasets.

### 2.6. Performance Indicators

Model performance was evaluated using a set of KPIs, which were calculated by comparing the network output against the first set of manual delineations as the ground truth on a per-pixel basis. The test dataset had a second set of manual delineations which were used to benchmark the results against a second human observer (the ‘2nd observer’). There were four potential classification outcomes for each pixel; true positive (TP), false positive (FP), true negative (TN) and false negative (FN). These outcomes were then used to derive KPIs, such as sensitivity (SN; also known as recall), specificity (SP), accuracy (Acc) and the receiver operating characteristic (ROC), which can be a function of SN and SP, true positive rate (TPR) and false positive rate (FPR), or other similar KPI pairs. SN and SP are two of the most important KPIs to consider when developing a classification system as they are both representations of the “truth condition” and are thereby a far better performance measure than Acc. In an ideal system, both SN and SP will be 100%, however this is rarely the case in real life. The area under a ROC curve (AUC), as well as Cohen’s kappa coefficient (κ), are two common approaches for measuring network performance. κ is measured using the probability ($n_{ki}$) of an observer (*i*) predicting a category (*k*) for a number of items (*N*) and provides a measure of agreement between observers—in this case, the network’s prediction and the ground truth [37].

The Matthews correlation coefficient (MCC), the F1-score (F1), and the G-mean (G) performance metrics were used to better assess the resulting fundus label masks. These particular metrics are well suited for cases with imbalanced class ratios, as with the abundance of non-vessel pixels comparative to a low number of vessel pixels in this binary segmentation task. MCC has been used to assess vessel segmentation performance in several cases, and its value is a range from −1 to +1, respectively indicating total disagreement or alignment between the ground truth and prediction [38]. Precision (Pr) is the proportion of positive samples properly classified and is often measured against SN in a precision-recall curve, similar to ROC. F-scores are harmonic means of Pr and SN, and may incorporate weightings to adjust for class imbalances. This work uses the F1-score with a range from 0 to 1, where 1 signifies perfect segmentation of the positive class. G-mean is the geometric mean between SN and SP. Importantly, G-mean is a better balance between SN and SP than AUC, making it a superior performance measure to AUC, as well as SN, SP and Pr individually [39]. The KPIs are defined in Table 1.

### 2.7. Training Details

For each dataset, the network parameters were randomly reinitialized using the Xavier algorithm [40]. Table 2 summarizes the three data sets as well as the test–train data distribution and approximate information loss incurred during preprocessing. Pre-training was never conducted and so the network was trained from scratch for each dataset. In the case of STARE and CHASE_DB1, one set of parameters was trained from scratch for each fold. Images were reduced in size to alleviate the computational burden of the training task rather than using the original image to train the network. Image size was first normalized to 256 × 256 before undergoing dataset augmentation. This step is the cause of the majority of information loss relative to the original images and, given the variance in dataset image size, was a convenient way to produce different degrees of information loss. Note, other methods compared herein extract patches rather than resize the original fundus images.

The images were randomly cropped to between 216 to 256 pixels along each axis and resized to 256 × 256. They were then randomly flipped both horizontally and vertically before being rotated at 0°, 90° or 180°. The brightness and contrast of each patch was randomly shifted to further increase network robustness. PixelBNN learned to generate vessel label masks from fundus images in batches of three for $1e^{5}$ iterations utilizing Adam optimization with an initial learning rate of $1e^{-5}$ and decay rate of 0.94 every $2e^{4}$ iterations. Batch normalization was conducted with an initial ϵ of $1e^{-5}$ and decay rate of 0.9 before the application of dropout regularization [41] with a keep probability of 0.6. It required approximately 11 h to complete training for DRIVE and the same for each fold during cross-validation.

## 3. Results

The output of PixelBNN was a binary label mask, predicting vessel and non-vessel pixels, thereby segmenting the original image. Each dataset contained manual delineations from two experts; the first was used as the ground truth for training the model and the second was used for evaluating the network’s performance against a secondary human observer. Independently, each dataset was used to train a separate model from scratch resulting in three sets of model parameters.

### 3.1. Performance Comparison

The results were compared with those of other state-of-the-art methods for vessel segmentation with published results for at least one of the DRIVE, STARE or CHASE_DB1 datasets. The results for the model trained and tested on DRIVE are shown in Table 3, STARE results are shown in Table 4 and CHASE_DB1 results are in Table 5. Cross-testing was conducted using each of these sets to measure the performance of the network against each other datasets’ test images. The results from cross-testing are summarized in Table 6. Most of the articles report SN and SP, relying on Acc and AUC to validate performance, whereas κ, MCC and F1-scores have been sparsely applied until recently. Regardless of other KPIs, most recent works report SN and SP from which the G-mean was calculated. Herein, the G-mean is considered to be a truer performance indicator than SN, SP and Pr. Further, the main KPIs used to evaluate model performance are F1-score, G-mean and MCC. For completeness, SN, SP, Pr, Acc, AUC and κ are also tabulated. Table 7 compares the computation time for training the network and evaluating test images with the methods that share the same GPU.

**Figure 2:**
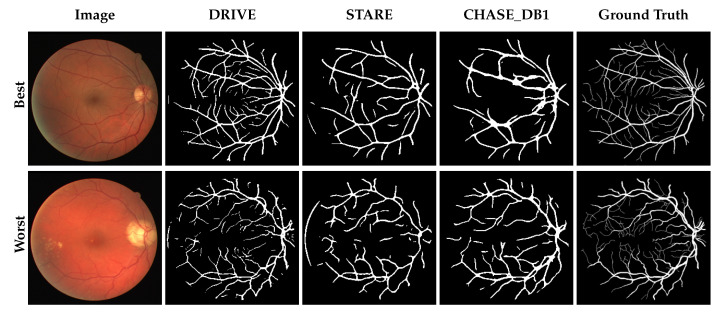
Network predictions on the DRIVE dataset. The top row shows the image, segmentation masks and ground truth for the image that scored best when DRIVE was used to train and test the model; the bottom row shows the worst. For comparison, the cross-validation results from training the model with STARE and CHASE_DB1 are shown.

**Figure 3:**
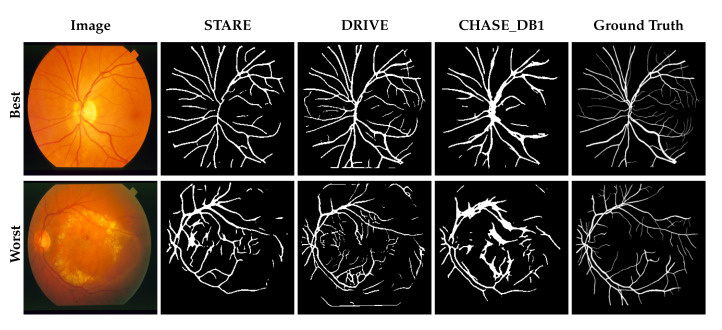
Network predictions on the STARE dataset. The top row shows the image, segmentation masks and ground truth for the image that scored best when STARE was used to train and test the model; the bottom row shows the worst. For comparison, the cross-validation results from training the model with DRIVE and CHASE_DB1 are shown.

**Figure 4:**
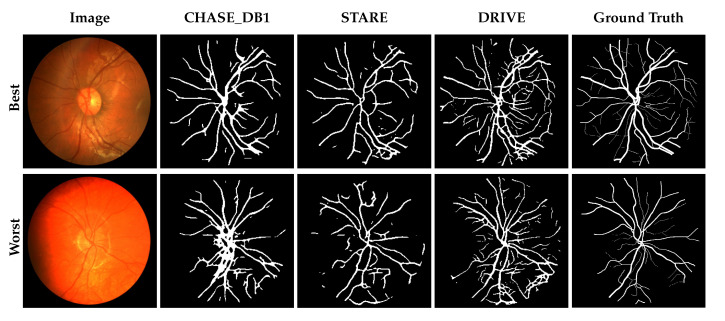
Network predictions on the CHASE_DB1 dataset. The top row shows the image, segmentation masks and ground truth for the image that scored best when CHASE_DB1 was used to train and test the model; the bottom row shows the worst. For comparison, the cross-validation results from training the model with STARE and DRIVE are shown.

Overall, the predictions reveal that losses in performance are largely the result of fine-vessels being missed as well as anomalous pathologies. Figure 2, Figure 3 and Figure 4 show the best and worst scoring same-set images, ground truth and resulting predictions for testing and cross-testing that image with DRIVE, STARE and CHASE_DB1 respectively. The model’s performance varied between datasets, outperforming other methods in a subset of cross-testing tasks for which there were few published baselines. At face value, the model appears to under-perform the state-of-the-art, however the information lost when resizing the images during preprocessing is quite severe.

### 3.2. Computation Time

Computation time is a difficult metric to benchmark due to variances in test system components and performance. In an attempt to evaluate this aspect, recent works that share the same GPU—the NVIDIA Titan X—were compared. This is a reasonable comparison, as the vast majority of computations are performed on the GPU when training DNNs. Table 7 shows the comparable methods’ approximate training and test speeds. Training time was evaluated by normalizing the total time for training the network by the number of training iterations. The total number of iterations was not provided in the multi-classifier article [55]. Test time is the duration required for evaluating one image at test time, end-to-end. The network evaluated test images in 0.0466 s, 8.6× faster than the state-of-the-art.

## 4. Discussion

Herein, the impact of information loss due to image resizing on performance and computational efficiency during vessel segmentation in fundus images was investigated. Different from the works in the literature, which use cropping and patch segmentation strategies, the proposed method instead resized the fundus images, shrinking them to 256 × 256. This incurred a loss of information as many pixels and details were discarded in the process, proportionately reducing the feature space by which the model could learn this task. The decision to explore this strategy was primarily driven by computational efficiency, as the methods are intended for use in real time within CAD systems in low-resource settings. A novel brain-inspired deep learning architecture was proposed for this task, coined PixelBNN as it is a variant of PixelCNN—a family of FCNs which has never before been applied to fundus images. DRIVE, STARE and CHASE_DB1 retinal fundus image datasets were used to evaluate model performance and generalizability across datasets. Compared to the other methods, PixelBNN used 5 × less information for DRIVE, 6.5× less for STARE, and 18.75× less information for CHASE_DB1 (see Table 2).

Basing the results of G-mean, MCC and F1-scores place the network performance in the middle of the back for DRIVE and STARE. The results are mixed for CHASE_DB1, as the G-mean is state-of-the-art, while the rest are quite poor. PixelBNN performed better on STARE and CHASE_DB1 when the model was trained with DRIVE rather than that same set, outperforming the state-of-the-art with regards to G-mean. The results show a loss of fine vessel detail, with SP degrading proportionately to information loss. This trend is not surprising, given deep learning method performance is dependant on the availability of data to train the system. Interestingly, SN follows this trend for DRIVE and STARE, but then increases dramatically with CHASE_DB1. The high degree of information loss results in over-merging vessel structures, resulting in state-of-the-art performance with regards to G-mean—its balance of SN to SP. Cross testing further exemplifies the heightened SN, and demonstrates the model’s ability to learn generalizable features even at severe levels of information loss.

Overall, the method showed an increase of 8.5× in computational efficiency versus the state-of-the-art, performed relatively well, even with a 19× reduction in information. Without further modification, this method may work well within larger CAD systems as an effective subroutine alongside specialized detection algorithms, or even in low-resource settings. It is worth noting that PixelBNN’s use is extensible to any image domain and its application to any task autoencoders can be applied to. Further refinement of the PixelBNN architecture and hyperparameters, such as increasing the number of streams or ResNets, may enable it as a standalone classifier. This will require delving into the architectural elements’ contributions as part of a generalized ablation study, which is left for future work.

## 5. Conclusions

This paper investigated the impact of information loss and computational efficiency due to image resizing, using PixelBNN on the task of vessel segmentation in retinal fundus images. This novel architecture performed well, even after a severe loss of information, outperforming state-of-the-art methods during cross-testing. It performed 8.5× faster than the current state-of-the-art, making it a viable candidate for application within real-world systems.

## Figures and Tables

**Table 1:** Key performance indicators.

KPI	Description	Value
True Positive Rate (TPR)	Probability of detection	$\frac{TP}{vessel\;pixel\;count}$
False Positive Rate (FPR)	Probability of false detection	$\frac{FP}{nonvessel\;pixel\;count}$
Accuracy (Acc)	The frequency a pixel is properly classified	$\frac{TP+TN}{total\;pixel\;count}$
Sensitivity aka Recall (SN)	The proportion of true positive results detected by the classifier	TPR or $\frac{TP}{TP+FN}$
Precision (Pr)	Proportion of positive samples properly classified	$\frac{TP}{TP+FP}$
Specificity (SP)	The proportion of negative samples properly classified	1−FPR or $\frac{TN}{TN+FP}$
Kappa (κ)	Agreement between two observers	$\frac{Acc-Acc_{prob}}{1-Acc_{prob}}$
Probability of Agreement ($Acc_{prob}$ )	Probability each observer $n_{ki}$ selects a category *k* for *N* items	$\frac{1}{N^{2}}\sum_{k}n_{k1}n_{k2}$
G-mean (G)	Balance measure of SN and SP	$\sqrt{SN*SP}$
F1 Score (F1)	Harmonic mean of precision and recall	$\frac{2*TP}{2TP+FP+FN}$ or $\frac{2*Pr*SN}{Pr+SN}$
Matthews correlation coefficient (MCC)	Measure from −1 to 1 of agreement between manual and predicted binary segmentations	$\frac{(TP/N)-S\times P}{\sqrt{P\times S\times (1-S)\times (1-P)}}$
		*N* = *TP* + *FP* + *TN* + *FN* *S* = *TP* + *FN* × *N* *P* = *TP* + *FP* × N

**Table 2:** Dataset statistics.

*Datasets*	DRIVE	STARE	CHASE_DB1
Image Dimensions	565 × 584	700 × 605	1280 × 960
Colour Channels	RGB	RGB	RGB
Total Images	40	20	28
Source Grouping	20 train and 20 test	-	14 Patients (2 images in each)
***Method Summary***			
Train—Test Schedule	One-off on 20 train, test on the other 20	4-fold cross-validation over 20 images	four-fold cross-validation over 14 patients
Information Loss	5.0348	6.4621	18.7500

**Table 3:** Performance comparison for models trained and tested with DRIVE.

Methods	SN	SP	Pr	Acc	AUC	kappa	G	MCC	F1
Human (2nd Observer)	0.7760	0.9730	0.8066	0.9472	-	0.7581	0.8689	0.7601	0.7881
***Unsupervised Methods***									
Lam et al. [42]	-	-	-	0.9472	0.9614	-	-	-	-
Azzopardi et al. [8]	0.7655	0.9704	-	0.9442	0.9614	-	0.8619	0.7475	-
Kovács and Hajdu [43]	0.7270	0.9877	-	0.9494	-	-	0.8474	-	-
Zhang et al. [44]	0.7743	0.9725	-	0.9476	0.9636	-	0.8678	-	-
Roychowdhury et al. [45]	0.7395± 0.062	0.9782± 0.0073	-	0.9494± 0.005	0.9672	-	0.8505	-	-
Niemeijer et al. [46]	0.6793± 0.0699	0.9801± 0.0085	-	0.9416± 0.0065	9294± 0.0152	0.7145	0.8160	-	-
***Supervised Methods***									
Soares et al. [10]	0.7332	0.9782	-	0.9461± 0.0058	0.9614	0.7285	0.8469	-	-
Ricci and Perfetti [3]	-	-	-	0.9595	0.9633	-	-	-	-
Marin et al. [47]	0.7067	0.9801	-	0.9452	0.9588	-	0.8322	-	-
Lupascu et al. [12]	-	-	-	0.9597± 0.0054	0.9561	0.7200	0.8151	-	-
Fraz et al. [48]	0.7152	0.9768	0.8205	0.9430	-	-	0.8358	0.7333	0.7642
Fraz et al. [7]	0.7406	0.9807	-	0.9480	0.9747	-	0.8522	-	-
Fraz et al. [49]	0.7302	0.9742	0.8112	0.9422	-	-	0.8434	0.7359	0.7686
Vega et al. [50]	0.7444	0.9600	-	0.9412	-	-	0.8454	0.6617	0.6884
Li et al. [51]	0.7569	0.9816	-	0.9527	0.9738	-	0.8620	-	-
Liskowski et al. [52]	0.7811	0.9807	-	0.9535	0.9790	0.7910	0.8752	-	-
Leopold et al. [53]	0.6823	0.9801	-	0.9419	0.9707	-	0.8178	-	-
Leopold et al. [54]	0.7800	0.9727	-	0.9478	0.9689	-	0.8710	-	-
Orlando et al. [38]	0.7897	0.9684	0.7854	-	-	-	0.8741	0.7556	0.7857
Mo et al. [55]	0.7779± 0.0849	0.9780± 0.0091	-	0.9521± 0.0057	0.9782± 0.0059	0.7759± 0.0329	0.8722± 0.0278	-	-
PixelBNN	0.6963± 0.0489	0.9573± 0.0089	0.7770± 0.0458	0.9106± 0.0121	0.8268± 0.0247	0.6795± 0.0414	0.8159± 0.0286	0.6820± 0.0399	0.7328± 0.0335

**Table 4:** Performance comparison for models trained and tested with STARE.

Methods	SN	SP	Pr	Acc	AUC	kappa	G	MCC	F1
Human (2nd Observer)	0.8951	0.9387	0.6424	0.9353	-	0.7046	0.9166	0.7225	0.7401
***Unsupervised Methods***									
Lam et al. [42]	-	-	-	0.9567	0.9739	-	-	-	-
Azzopardi et al. [8]	0.7716	0.9701	-	0.9497	0.9563	-	0.8652	0.7335	-
Kovács and Hajdu [43]	0.7665	0.9879	-	-	0.9711	-	0.8702	-	-
Zhang et al. [44]	0.7791	0.9758	-	0.9554	0.9748	-	0.8719	-	-
Roychowdhury et al. [45]	0.7317± 0.053	0.9842± 0.0069	-	0.9560± 0.0095	0.9673	-	0.8486± 0.0178	-	-
***Supervised Methods***									
Soares et al. [10]	0.7207	0.9747	-	0.9479	0.9671	-	0.8381	-	-
Ricci et al. [3]	-	-	-	0.9584	0.9602	-	-	-	-
Marin et al. [47]	0.6944	0.9819	-	0.9526	0.9769	-	0.8257	-	-
Fraz et al. [48]	0.7409	0.9665	0.7363	0.9437	-	-	0.8462	0.7003	0.7386
Fraz et al. [7]	0.7548	0.9763	-	0.9534	0.9768	-	0.8584	-	-
Fraz et al. [49]	0.7318	0.9660	0.7294	0.9423	-	-	0.8408	0.6908	0.7306
Vega et al. [50]	0.7019	0.9671	-	0.9483	-	-	0.8239	0.5927	0.6082
Li et al. [51]	0.7726	0.9844	-	0.9628	0.9879	-	0.8721	-	-
Liskowski et al. [52]	0.8554± 0.0286	0.9862± 0.0018	-	0.9729± 0.0027	0.9928± 0.0014	0.8507± 0.0155	0.9185± 0.0072	-	-
Mo et al. [55]	0.8147± 0.0387	0.9844± 0.0034	-	0.9674± 0.0058	0.9885± 0.0035	0.8163± 0.0310	0.8955± 0.0115	-	-
Orlando et al. [38]	0.7680	0.9738	0.7740	-	-	-	0.8628	0.7417	0.7644
PixelBNN	0.6433± 0.0593	0.9472± 0.0212	0.6637± 0.1135	0.9045± 0.0207	0.7952± 0.0315	0.5918± 0.0721	0.7797± 0.0371	0.5960± 0.0719	0.6465± 0.0621

**Table 5:** Performance comparison for models trained and tested with CHASE_DB1.

Methods	SN	SP	Pr	Acc	AUC	kappa	G	MCC	F1
Human (2nd Observer)	0.7425	0.9793	0.8090	0.9560	-	0.7529	0.8527	0.7475	0.7686
***Unsupervised Methods***									
Azzopardi et al. [8]	0.7585	0.9587	-	0.9387	0.9487	-	0.8527	0.6802	-
Zhang et al. [44]	0.7626	0.9661	-	0.9452	0.9606	-	0.8583	-	-
Roychowdhury et al. [45]	0.7615± 0.0516	0.9575± 0.003	-	0.9467± 0.0076	0.9623	-	0.8539± 0.0124	-	-
***Supervised Methods***									
Fraz et al. [7]	0.7224	0.9711	-	0.9469	0.9712	-	0.8376	-	-
Li et al. [51]	0.7507	0.9793	-	0.9581	0.9716	-	0.8574	-	-
Liskowski et al. [52]	0.7816± 0.0178	0.9836± 0.0022	-	0.9628± 0.0020	0.9823± 0.0016	0.7908± 0.0111	0.8768± 0.0063	-	-
Mo et al. [55]	0.7661 ± 0.0533	0.9816± 0.0076	-	0.9599± 0.0050	0.9812± 0.0040	0.8672± 0.0201	0.7689± 0.0263	-	-
Orlando et al. [38]	0.7277	0.9712	0.7438	-	-	-	0.8403	0.7046	0.7332
PixelBNN	0.8618± 0.0232	0.8961± 0.0150	0.3951± 0.0603	0.8936± 0.0138	0.878959± 0.0138	0.4889± 0.0609	0.8787± 0.0140	0.5376± 0.0491	0.5391± 0.0587

**Table 6:** Model performance measures from cross-training.

Methods			SN	SP	Pr	Acc	AUC	kappa	G	MCC	F1
*Test images from: **DRIVE***										
*Model* *trained on:* **STARE**	Soares et al. [10]	-	-	-	0.9397	-	-	-	-	-
	Ricci et al. [3]	-	-	-	0.9266	-	-	-	-	-
	Marin et al. [47]	-	-	-	0.9448	-	-	-	-	-
	Fraz et al. [7]	0.7242	0.9792	-	0.9456	0.9697	-	0.8421	-	-
	Li et al. [51]	0.7273	0.9810	-	0.9486	0.9677	-	0.8447	-	-
	Liskowski et al. [52]	-	-	-	0.9416	0.9605	-	-	-	-
	Mo et al. [55]	0.7412	0.9799	-	0.9492	0.9653	-	0.8522	-	-
	*PixelBNN*	0.5110± 0.0362	0.9533± 0.0094	0.7087± 0.0554	0.8748± 0.0126	0.7322± 0.0199	0.5193± 0.0404	0.6974± 0.0258	0.5309± 0.0422	0.5907± 0.0348
*Model* *trained on:* **CHASE_DB1**	Li et al. [51]	0.7307	0.9811	-	0.9484	0.9605	-	0.8467	-	-
	Mo et al. [55]	0.7315	0.9778	-	0.9460	0.9650	-	0.8457	-	-
	PixelBNN	0.6222± 0.0441	0.9355± 0.0085	0.6785± 0.0383	0.8796± 0.0090	0.7788± 0.0204	0.5742± 0.0282	0.7622± 0.0254	0.5768± 0.0279	0.6463± 0.0237
*Test images from: **STARE***										
*Model* *trained on:* **DRIVE**	Soares et al. [10]	-	-	-	0.9327	-	-	-	-	-
	Ricci et al. [3]	-	-	-	0.9464	-	-	-	-	-
	Marin et al. [47]	-	-	-	0.9528	-	-	-	-	-
	Fraz et al. [7]	0.7010	0.9770	-	0.9493	0.9660	-	0.8276	-	-
	Li et al. [51]	0.7027	0.9828	-	0.9545	0.9671	-	0.8310	-	-
	Liskowski et al. [52]	-	-	-	0.9505	0.9595	-	-	-	-
	Mo et al. [55]	0.7009	0.9843	-	0.9570	0.9751	-	0.8306	-	-
	PixelBNN	0.7842± 0.0552	0.9265± 0.0196	0.6262± 0.1143	0.9070± 0.0181	0.8553± 0.0323	0.6383± 0.0942	0.8519± 0.0343	0.6465± 0.0873	0.6916± 0.0868
*Model* *trained on:* **CHASE_DB1**	Li et al. [51]	0.6944	0.9831	-	0.9536	0.9620	-	0.8262	-	-
	Mo et al. [55]	0.7387	0.9787	-	0.9549	0.9781	-	0.8503	-	-
	PixelBNN	0.6973± 0.0372	0.9062± 0.0189	0.5447± 0.0957	0.8771± 0.0157	0.8017± 0.0226	0.5353± 0.0718	0.7941± 0.0245	0.5441± 0.0649	0.6057± 0.0674
*Test images from: **CHASE_DB1***										
*Model* *trained on:* **DRIVE**	Li et al. [51]	0.7118	0.9791	-	0.9429	0.9628	-	0.8348	-	-
	Mo et al. [55]	0.7003	0.9750	-	0.9478	0.9671	-	0.8263	-	-
	PixelBNN	0.9038± 0.0196	0.8891± 0.0089	0.3886± 0.0504	0.8901± 0.0088	0.8964± 0.0116	0.4906± 0.0516	0.8963± 0.0116	0.5480± 0.0413	0.5416± 0.0513
*Model* *trained on:* **STARE**	Fraz et al. [7]	0.7103	0.9665	-	0.9415	0.9565	-	0.8286	-	-
	Li et al. [51]	0.7240	0.9768	-	0.9417	0.9553	-	0.8410	-	-
	Mo et al. [55]	0.7032	0.9794	-	0.9515	0.9690	-	0.8299	-	-
	PixelBNN	0.7525± 0.0233	0.9302± 0.0066	0.4619± 0.0570	0.9173± 0.0059	0.8413± 0.0132	0.5266± 0.0482	0.8365± 0.0143	0.5475± 0.0412	0.5688± 0.0475

**Table 7:** Computation times for different networks using an NVIDIA Titan X.

Method	Description	Training Time (s/iteration)	Test Time (s/image)
Liskowski et al. [52]	Repurposed MNIST LeNet	0.96	92
Mo et al. [55]	Pre-trained Multi-classifier	*N/A*	0.4
PixelBNN	Proposed Method	0.52	0.0466
